# Allometric normalization of handgrip strength in older adults: Which body size parameter is the most appropriate?

**DOI:** 10.1186/s13102-023-00628-0

**Published:** 2023-02-08

**Authors:** Mario Kasović, Peter Sagat, Zvonimir Kalčik, Lovro Štefan, Andrej Hubinák, Peter Krška

**Affiliations:** 1 Department of General and Applied Kinesiology, Faculty of Kinesiology, University of Zagreb, Horvaćanski zavoj 15, 10 000 Zagreb, Croatia; 2 Department of Physical Activities and Health Sciences, Faculty of Sports Studies, Masaryk University, Brno, Czech Republic; 3 Albert Einstein, Bc., Prince Sultan University, Sport Sciences and Diagnostics Research Group, GSD-HPE Department, Riyadh, Saudi Arabia; 4 The Home of War Veterans, Zagreb, Croatia; 5 Recruitment and Examination (RECETOX), Faculty of Science, Masaryk University, Brno, Czech Republic; 6 Department of Physical Education and Sport, Faculty of Education, Catholic University in Ružomberok, Ružomberok, Slovakia

**Keywords:** Allometric modelling, Elderly population, Body size, Muscle strength dynamometer performance, Hand strength

## Abstract

**Background:**

Although absolute handgrip strength has been associated with health-related outcomes in older adults, little evidence has been provided regarding its adjustment by a variety of body size dimensions. Therefore, the main purpose of the study was to establish the most appropriate normalization of handgrip strength by different body size parameters in a large sample of noninstitutionalized older adults.

**Methods:**

In this cross-sectional study, we enrolled 643 men and women aged > 60, who were part of the rehabilitation center facility program. Handgrip strength was objectively measured using a Jamar Plus* + Digital Hand Dynamometer. Body size parameters included body weight and height, body mass index, waist circumference, waist-to-height ratio, fat mass and fat-free mass. The most appropriate parameter associated with handgrip strength was identified using allometry.

**Results:**

Findings showed that the most appropriate body size parameter for handgrip strength normalization was height (allometric exponent: 0.85), compared to fat-free mass (0.26) and body mass (0.12). Other body size variables were not significantly associated with handgrip strength and were omitted from further analyses. The correlations between normalized handgrip strength were significant when handgrip strength was normalized by body mass and fat-free mass, while no significant correlations were found, when handgrip strength was normalized by body height.

**Conclusion:**

Based on the study results, body height seems to be the best body size parameter for handgrip strength normalization in older adults, omitting the influence of body size on strength performance. If handgrip strength is measured, body height may help normalize strength for large-scale research.

## Introduction

Aging process is associated with a reduction of muscle strength [1]. Muscle strength is considered a key element for physical performance and independence in older adults [2]. Evidence shows that lower level of muscle strength leads to higher incidence of cardiovascular [3], metabolic [4], and cerebrovascular diseases [5], falls [6], early hospitalization [7] and premature mortality [8].

There are many alternatives for assessing muscle strength in older adults which include tools and methods that measure voluntary movements related to strength [9]. Handgrip strength is a simple and easily utilized surrogate of the maximum voluntary contraction force of the hand for the assessment of muscle function [9, 10]. Low handgrip strength has been associated with several poor health-related outcomes such as functional deficits, cognitive declines, chronic morbidity and premature mortality [11]. Thus, its applicability has become important in clinical and epidemiological settings.

The measure of handgrip strength is often presented as an absolute (non-normalized) value. This would imply that larger individuals would have an advantage over smaller ones, if the results are not correctly normalized [12]. To overcome this problem, previous evidence in older adults has proposed that the ratio of the absolute handgrip strength should be normalized by different body size parameters such as body weight [11, 13–17], body height [13, 14, 17], body mass index [13, 15], fat-free mass [14] and waist circumference [17]. This approach is usually called ‘allometric scaling’ and represents the method used for taking into account the body size effect [18, 19]. As one would expect, the most common variable used in allometric adjustments is body mass [16]. However, little is known about different body size parameters being compared to assess the most appropriate variable to be used in the normalization model of handgrip strength in older adults [14]. The most recent study by Nevill et al. [17] showed that neither body mass nor body mass index were appropriate to normalize handgrip strength, yet height was shown to be the best body dimension associated with handgrip strength. Also, only a small number of previous studies have used sex [14, 20] and age [14, 21] in the allometric models, although their influence has been well described in the literature.

Therefore, the main purpose of the study was to establish the most appropriate body size dimension by comparing the effects of body weight, body height, body mass index, waist circumference, waist-to-height ratio, fat mass and fat-free mass to normalize handgrip strength in a large sample of community elderly. Based on previous evidence [14, 17], we hypothesized that body height would be more appropriate than other variables for being used on the isolation of body size influence.

## Materials and methods

### Study participants

Our sample size was based on measurements taken from 2020 to 2022 for 1000 participants. The inclusion criteria for participation in the study included a self-reported information on: (i) being without chronic diseases, which included chronic heart disease, rheumatic arthritis, diabetes, chronic kidney disease, stroke, cancer and chronic obstructive pulmonary disease, (ii) the absence of a serious physical or mental illness, and (iii) having all the study variables tested. Of 1000 participants, 840 met the inclusion criteria. The participants, who were excluded from further analyses suffered from chronic heart diseases (45%), rheumatic arthritis (4%), cancer (2%), chronic obstructive pulmonary disease (15%) and diabetes (11%). After re-analysis, 643 men and women had all the study variable measurements and were free from SARS-CoV infection at the time of measurement. Before data collection started, all participants were informed about the aim, hypotheses and methodology of the study. The participants were ensured confidentiality and informed that their participation was voluntary, and that they had the right to withdraw at any time. All participants have read and signed the informed consent forms. We followed the methods of the principles of the Declaration of Helsinki [22], and the Ethical Committee of The Home of War Veterans approved the study (Ethical code number: 2022/4).

### Handgrip strength measurement

To assess handgrip strength, we used a Jamar Plus* + Digital Hand Dynamometer (Sammons Preston Inc., Bolingbrook, Illinois, USA). The device was calibrated by the manufacturer, with a precision of 0.1 kg. The protocol for measuring handgrip strength was standardized and followed by the American Society of Hand Therapists [23]. In brief, the participant was placed in a seated position with shoulder rotated and adducted in a neutral position, forearm in neutral position, elbow flexed at 90° and wrist between 0 and 30° of dorsiflexion. Each participant conducted the measurement three times (with a 60 s rest interval between attempts to avoid fatigue) with the non-dominant hand [23]. Previous meta-analysis has shown no significant differences and trivial effect sizes between the dominant and the non-dominant hand [24]. Out of three measurements, the best one was recorded and used in further analyses [14]. The coefficient of variation between the trials was 4.5%, which corresponded to approximately 1.5 kg and the analysis of variance (ANOVA) revealed no significant differences (*p* = 0.548). The additional time was considered, if the participant reported fatigue. A 5 min warm-up was implemented before the testing and it was supervised by a trained coach. During the measurement, the participants were verbally motivated to continue their maximum strength and to perform all repetitions.

### Anthropometric measurement

Body height and weight were objectively measured using Seca portable stadiometer and digital scale with a precision of 0.1 cm and 0.1 kg. Body height was measured in bare or stocking feet standing upright against a stadiometer and body weight with wearing light clothes with no shoes. Body mass index was calculated (weight [kg]/height [m]^2^). Waist circumference was measured using anthropometric tape placed horizontally midway between the lower rib margin and the iliac crest at the end of normal expiration, while the participant was standing still [25]. Waist-to-height ratio was calculated by dividing waist circumference with body height (in cm). To assess fat mass and fat-free mass, we used bioelectrical impedance analysis (Omron BF500 Body Composition Monitor, Omron Medizintechnik, Vernon Hills, IL, USA). The device uses eight electrodes and pre-programmed equations to determine fat mass. The participant was required to stand on metal footpads barefoot and grasp a pair of electrodes fixed on a handle with arms extended in front of the chest [26]. Sex and age were self-reported.

### Statistical analysis

The statistical analyses were conducted using Statistical Packages for Social Sciences version 23 (SPSS Inc., Chicago, IL, USA). Descriptive statistics were calculated for all variables. Continuous variables were presented as means and standard deviations (SD). Sex differences were examined with Student *t*-test for independent samples. The magnitude of the differences between the sexes in each variable was calculated using Cohen’s *D* effect size (ES). According to Hopkins et al. [27], ES was classified as trivial (< 0.2), small (0.2–0.6), moderate (0.6–1.2), large (1.2–2.0), very large (> 2.0) and extremely large (> 4.0). An allometric scaling was done by including the natural logarithm (*ln*) of each body size dimension as the independent variable against the natural logarithm of handgrip strength as the dependent variable into the log-regression model. Sex (1 for men and 2 for women) and age (a continuous variable) served as covariates in each model. In final analysis, ln body size*sex*age interaction terms were entered and if the interaction terms were not statistically significant, they would be excluded. When the regression equation for each body size and handgrip strength was determined, we tested the data for multicollinearity using the variance inflation factors, normality of residuals using the normal probability plot and histogram of residuals and heteroscedasticity using the standardized residuals versus predicted plot. The variance inflation factors in our model ranged from 1.03 to 2.12 indicating no multicollinearity and the other assumptions were also met. Next, a Pearson correlation analysis was performed between the body size variables and normalized handgrip strength. If the adjustments were significant (*p* < 0.05), the model would be considered inadequate, because the model was incapable of isolating the body size effect on the handgrip strength. Two-sided *p*-values were used, and significance was set at α < 0.05.

## Results

The Kolmogorov–Smirnov test showed that all study variables were normally distributed. Basic descriptive statistics of the study participants according to sex are presented in Table 1. Men were taller, heavier and had higher body mass index and fat-free mass values, compared to women (*p* < 0.05). Women exhibited lower waist circumference, but higher fat mass values, compared to men (*p* < 0.05). The largest effects in absolute handgrip strength were observed, where men generated ≈ 35.0% higher values.

**Table 1:** Basic descriptive statistics of the study participants presented as mean (SD)

Study variables	Men (*N* = 260)	Women (*N* = 383)	ES	*p* for sex
Age (years)	67.4 (5.5)	66.9 (5.2)	0.09	0.160
Height (cm)	172.9 (5.0)	161.1 (6.0)	2.14	< 0.001
Weight (kg)	84.0 (10.3)	70.0 (12.1)	1.25	< 0.001
Body mass index (kg/m^2^)	27.7 (3.3)	26.9 (4.2)	0.21	0.027
Waist circumference (cm)	100.1 (9.3)	90.5 (11.6)	0.91	< 0.001
Waist-to-height ratio	0.58 (0.1)	0.56 (0.1)	0.20	0.033
Fat mass (%)	31.2 (7.0)	38.2 (6.6)	1.03	< 0.001
Fat-free mass (%)	70.9 (4.6)	61.8 (6.6)	1.60	< 0.001
Handgrip strength (kg)	46.9 (7.6)	30.5 (5.3)	2.50	< 0.001

*ES* Effect size; *p* < 0.05

Table 2 shows regression coefficients for the log-transformed independent variables. The separate interaction terms representing the associations between ln handgrip strength with ln body mass index (*p* = 0.099), ln waist circumference (*p* = 0.367), ln waist-to-height ratio (*p* = 0.067) and ln fat mass (*p* = 0.078) were not statistically significant, so we omitted them from further analyses. The final three regression models were based on body mass, body height and fat-free mass additionally adjusted for sex and age. Allometric adjustments for each model were generated, where β coefficient served as an exponent. The obtained exponents were 0.12 for body mass, 0.85 for body height and 0.26 for fat-free mass. The following equation using the exponents should be applied: “adjusted handgrip strength = absolute handgrip strength (kg)/body size variable^exponent^”.

**Table 2:** Allometric scaling of handgrip strength and body size variables, adjusting for sex and age (*N* = 643)

Study variables	β (SE)	95% CI	*p*-value	VIF	R	R^2^
Model 1						
Constant	1.92 (0.12)	1.69 to 2.16	< 0.001		0.79	0.63
Ln body mass	0.12 (0.05)	0.01 to 0.22	0.029	1.38		
Sex	− 0.19 (0.01)	− 0.20 to − 0.17	< 0.001	1.41		
Age	− 0.004 (0.001)	− 0.006 to − 0.003	< 0.001	1.03		
Model 2						
Constant	1.65 (0.08)	1.50 to 1.81	< 0.001		0.82	0.67
Ln body height	0.85 (0.10)	0.64 to 1.05	< 0.001	2.08		
Sex	− 0.14 (0.01)	− 0.16 to − 0.12	< 0.001	2.12		
Age	− 0.004 (0.001)	− 0.005 to − 0.003	< 0.001	1.04		
Model 3						
Constant	1.66 (0.17)	1.33 to 1.99	< 0.001		0.80	0.64
Ln fat free mass	0.26 (0.08)	0.10 to 0.43	0.002	1.51		
Sex	− 0.18 (0.009)	− 0.20 to − 0.16	< 0.001	1.54		
Age	− 0.004 (0.001)	− 0.006 to − 0.003	< 0.001	1.03		

*β* Unstandardized beta coefficient, *SE* Standard error, *95% CI* 95 percent confidence interval, *VIF* Variance inflation index, *R* Coefficient of correlation, *R*^*2*^ Coefficient of determination, *Ln* Logarithm exponent; *p* < 0.05

The correlations between adjusted handgrip strength and body size variables are presented in Table 3. When the handgrip strength was normalized by body mass, small but significant correlations with height and fat-free mass were observed (*p* < 0.05). Similar effects were shown for handgrip strength normalized by fat-free mass and body weight and height (*p* < 0.05). However, the correlations between normalized handgrip strength by body height and the body size variables were not statistically significant, implicating that this model might be adequate for mitigating the body size effect on the handgrip strength.

**Table 3:** Correlations between adjusted handgrip strength with body size variables, adjusted for sex and age

Study variables	Pearson’s r	*p*-value	ES
Handgrip strength/body mass^0.12^			
Body mass (kg)	− 0.002	0.965	Trivial
Body height (cm)	0.32	< 0.001	Small
Fat-free mass (%)	0.24	< 0.001	Small
Handgrip strength/body height^0.85^			
Body mass (kg)	− 0.04	0.468	Trivial
Body height (cm)	− 0.004	0.939	Trivial
Fat-free mass (%)	0.01	0.847	Trivial
Handgrip strength/fat-free mass^0.26^			
Body mass (kg)	0.22	< 0.001	Small
Body height (cm)	0.26	< 0.001	Small
Fat-free mass (%)	0.006	0.896	Trivial

*ES* Effect size;* p* < 0.05

## Discussion

The main purpose of the study was to examine and compare the performance of different body size dimensions (body weight, body height, body mass index, waist circumference, waist-to-height ratio and fat-free mass) in the normalization of handgrip strength and adjusted for sex and age in a large sample of older adults. Our main findings are: (i) body height is the most appropriate body size parameters for being used in allometric scaling of handgrip strength, followed by fat-free mass and body weight; (ii) the interaction terms between body mass index, waist circumference, waist-to-height ratio and fat mass with handgrip strength are not significant; and (iii) body mass and fat-free mass show residual associations with allometrically normalized handgrip strength, pointing out that these variable are incapable to completely exclude the body size effect on the handgrip strength.

Evidence recommends that handgrip strength should be normalized for body size dimensions [9, 12]. The most common body size dimensions include weight [11, 13–17], body height [13, 14, 17], body mass index [13, 15], fat-free mass [14] and waist circumference [17]. Our finding of body mass exponent being 0.12 is smaller, compared to other studies conducted in older adults [12, 14, 16, 28]. For instance, a study by Pua [16] showed larger exponents for body mass and handgrip force (0.63), ankle dorsiflexion force (0.82), ankle dorsiflexion torque (0.91) and Timed “Up & Go” Test (0.07). A study by Maranhao Neto et al*.* [14] found that body mass exponent for handgrip strength was 0.31, while Foley et al*.* [28] reported 0.40 in the body mass exponent for handgrip strength allometric normalization. Regarding fat-free mass, our results showed the exponent of 0.26, which is lower than those obtained in previous studies [14, 29]. Indeed, Folland et al. [29] presented the findings, where fat-free mass exhibited higher exponents for force (0.76) and torque (1.12) and determined on average 54.0% of the variation in knee extensor torque. When the regression model was adjusted for sex, age and the level of physical activity, Maranhao Neto et al*.* [14] found 0.46 in the fat-free mass exponent. Our results obtained in the body height exponent (0.85) were like previous findings of 0.92 [29] and 1.1 [30]. However, higher body height exponent of 1.84 was demonstrated for Brazilian older adults [14]. The discrepancy between the exponents for different body size parameters may be due to different sample size, wide age range, adjustment for specific variables and testing measurements of handgrip strength, not being able to generalize the findings and its representativeness. Although we followed the recommendations from the American Society of Hand Therapists for testing handgrip strength [23], previous protocols have used the handgrip strength measured with the elbow in extension [14]. Nevertheless, this is the first study comparing a variety of body size parameters in relation to absolute handgrip strength and adjusting for sex and age in a relatively large sample of older adults.

Greater muscle strength is an important component of preserving from cardiometabolic, locomotor and mental diseases [11]. While the applicability of testing handgrip strength and its normalization has been well-documented in the literature [9, 10], studies often fail to adjust for body size parameters with the misleading results. This study has shown that by using body height as the most appropriate body size dimension for allometric scaling, it is possible to provide an unbiased body size adjustment in elderly population.

This study has several limitations. First, by using a cross-sectional design, we cannot determine the causality of the association between body size dimensions and handgrip strength. Second, to secure a homogenous sample on which allometric scaling was performed, this study was limited to a sample of free-living community-dwelling older adults without locomotor and mental diseases and who did not fall in the past year. Third, although we included fat mass and fat-free mass as body size dimensions, we used bioelectrical impedance analysis and more objective methods, like DEXA or computer tomography might have given different exponents. Fourth, the results of maximal testing are co-dependent with the maximal effort of participants, and because of the nature of the study, this cannot be fully guaranteed. Fifth, the data regarding the level of physical activity and history of hospitalization were not collected, limiting the generalizability of our findings. Finally, the results of our study should be conducted in older adults suffering from physical and mental diseases, in order to broaden up the findings to other populations.

The handgrip strength measurement is an important fitness tool to assess functionality and performance of upper limbs, especially in community-dwelling older adults. However, by linking handgrip strength to current or future health, previous studies have used inappropriate scaling approaches like body mass or body-mass index, that cannot fully diminish the effect of body size on handgrip strength values. Since evidence has been inconclusive for handgrip strength normalization, our findings suggest that body height can be the most appropriate body size parameter, which fully omits the effect of body size on handgrip strength results. Second, future reports should state raw handgrip strength values, in order for findings to be comparable between the studies. Third, we were unable to compare the findings to those individuals, who suffered from diseases. This may imply that the type of a disease, the level of physical activity or previous history of hospitalization can act as confounding variables and affect the internal validity of the study. Finally, reference charts representing percentiles should be sex- and age-specific, in order to understand an individual’s rank and detect a ‘risky’ group of community-dwelling older adults, who are at increased risk for having low handgrip strength values. For better understanding, previous studies have recommended a quintile categorization from “very low” (< 20th percentile), “low” (20–40th percentile), “average” (40–60th percentile), “high” (60–80th percentile) and “very high” (> 80th percentile) handgrip strength.

## Conclusion

This study suggests that among various body size parameters, body height seems to be the most appropriate allometric variable which completely remove the effect of body size on muscle strength performance in community-dwelling older adults. By using a relatively large sample size, we were able to generate the body exponential value of 0.85 for normalization of handgrip strength. Thus, the following equations should be considered in clinical and epidemiological settings in similar populations: “corrected handgrip strength = absolute handgrip strength (kg)/body height (m^0.85^)”. The newly developed allometric equation should be used in community-dwelling older adults when testing handgrip strength.

## Data Availability

The datasets used and/or analyzed during the current study are available from the corresponding author on reasonable request.
